# Emerging Trends in Microfluidic Biomaterials: From Functional Design to Applications

**DOI:** 10.3390/jfb16050166

**Published:** 2025-05-08

**Authors:** Jiaqi Lin, Lijuan Cui, Xiaokun Shi, Shuping Wu

**Affiliations:** Institute of Polymer Materials, School of Materials Science & Engineering, Jiangsu University, Zhenjiang 212013, China; 3210708135@stmail.ujs.edu.cn (J.L.); 2222305061@stmail.ujs.edu.cn (L.C.); 2212405057@stmail.ujs.edu.cn (X.S.)

**Keywords:** microfluidic technology, microfluidic biomaterials, organ-on-a-chip, 3D bioprinting, medical applications

## Abstract

The rapid development of microfluidics has driven innovations in material engineering, particularly through its ability to precisely manipulate fluids and cells at microscopic scales. Microfluidic biomaterials, a cutting-edge interdisciplinary field integrating microfluidic technology with biomaterials science, are revolutionizing biomedical research. This review focuses on the functional design and fabrication of organ-on-a-chip (OoAC) platforms via 3D bioprinting, explores the applications of biomaterials in drug delivery, cell culture, and tissue engineering, and evaluates the potential of microfluidic systems in advancing personalized healthcare. We systematically analyze the evolution of microfluidic materials—from silicon and glass to polymers and paper—and highlight the advantages of 3D bioprinting over traditional fabrication methods. Currently, despite significant advances in microfluidics in medicine, challenges in scalability, stability, and clinical translation remain. The future of microfluidic biomaterials will depend on combining 3D bioprinting with dynamic functional design, developing hybrid strategies that combine traditional molds with bio-printed structures, and using artificial intelligence to monitor drug delivery or tissue response in real time. We believe that interdisciplinary collaborations between materials science, micromachining, and clinical medicine will accelerate the translation of organ-on-a-chip platforms into personalized therapies and high-throughput drug screening tools.

## 1. Introduction

**Figure 1:**
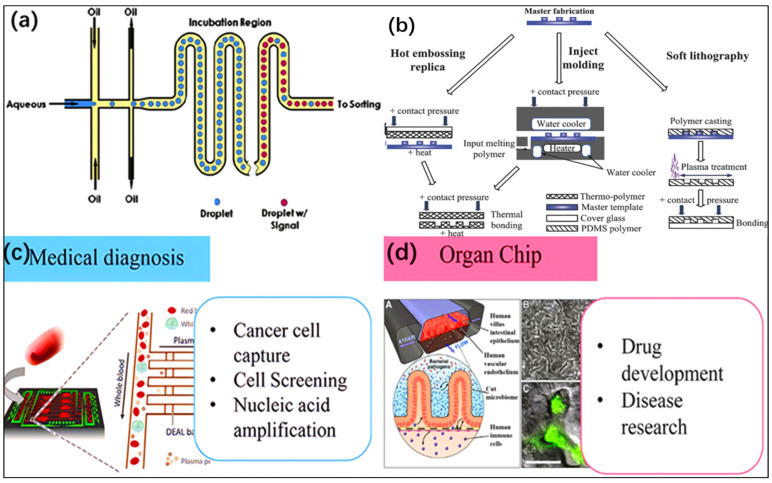
(**a**) Conceptual diagram of microfluidic technology. This diagram depicts the process of mixing the water phase with the oil phase together through micro/nanoscale channels to form tiny droplets. (**b**) Typical manufacturing processes of hot pressing, injection molding, and soft lithography methods (reprinted from Ref. [9]). (**c**,**d**) Examples of microfluidic chips used in medical diagnosis and organ chips (reprinted from Ref. [33]).

Microfluidics, which refers to fluids flowing in channels ranging in size from a hundred nanometers to several hundred micrometers, is an emerging interdisciplinary technique that manipulates single-phase or multiphase fluids at the microscale [1,2,3]. The flow is dominated by interfacial tension, viscous dissipation, and fluid resistance. During the time period of the 1990s, the field of microfluidics gained great appeal. Over the past three decades, microfluidic technology has experienced explosive development (Figure 1a). In the selection of microfluidic materials, silicon and glass were initially used, but later the focus was mainly on polymer materials, especially polydimethylsiloxane (PDMS). In recent years, paper materials have come to the forefront and have achieved significant results in some applications [4,5,6,7]. Since then, the field of microfluidics has evolved to include various materials and applications. It has also evolved to meet individual needs in different situations. Several manufacturing processes have been used to create microfluidic devices. Traditional methods for manufacturing microfluidic devices are prototype technologies, mainly including hot pressing [8], injection molding, and soft lithography [9,10,11] (Figure 1b). However, with the advancement of technology, 3D printing is becoming increasingly common [12,13,14,15].

Microfluidic biomaterials are the most successful and widely used microfluidic technology. In recent years, microfluidic technology has been widely applied in the field of biomaterials [16,17,18]. The term biomaterials refer to materials that are used to interact with biological systems for diagnosis, drug therapy, cell repair, or replacement of tissues, organs, or functions [19,20,21,22]. Their core advantage lies in their ability to achieve efficient and controllable fluid manipulation in a very small volume, providing a new platform for the synthesis, characterization, and application of biomaterials. The high throughput, low consumption, and precise control characteristics of microfluidic systems make them an important tool in biomaterial research—especially in fields such as drug delivery [23,24,25], tissue engineering [26,27,28], and cell culture [29,30,31]—demonstrating enormous potential. In summary, microfluidic biomaterials combine the precise manipulation capability of microfluidic technology with the biocompatibility of biomaterials and thus show tremendous potential for application in the field of biomedicine. With the cross-integration of materials science, microfabrication technology, and biomedical research, the study of microfluidic biomaterials is rapidly advancing from basic functional design to practical applications, becoming an important driving force for the development of biomedical engineering.

Among many microfluidic biological devices, microfluidic chips have unique advantages. Microfluidic chips have a small volume, high mass and heat transfer efficiency, and fast reaction speed at the microscale, making them suitable for on-site detection and portable devices. They are widely used in medical diagnosis [32] and organ chips [33] (Figure 1b,c). Among them, organ-on-a-chip (OoAC) devices use microfluidic technology to accurately replicate the in vivo environment, especially to simulate the human cellular microenvironment. It stimulates the vascular system network by flowing fluid through microchannels to provide nutrients and transport waste and metabolites [34,35,36]. The microfluidic channels provide a basis for creating a physiologically similar environment to culture the organ cells of interest [37,38,39]. OoAC devices have a wide range of applications in the medical field. They can mimic the structure and function of human organs, and the physiological environment of replicating specific organs can be further subdivided into simulated diseases based on their respective modeled organs [40,41,42]. The OoAC platform also provides more accurate models for studying human biology and disease, offering opportunities for personalized medicine and tailored therapies for specific patients or populations [43,44,45,46].

At the application level, microfluidic biomaterials are evolving toward multifunctionality, intelligence, and integration. For example, intelligent drug delivery systems based on microfluidic technology can achieve precise drug release, while microfluidic tissue engineering platforms provide the ability to construct complex 3D tissue models [47,48,49,50,51]. In addition, the application of microfluidic technology in real-time diagnostics and portable medical devices has shown great promise. However, despite significant progress in microfluidic biomaterials, they still face many challenges in terms of large-scale production, long-term stability, and clinical translation [52,53,54,55].

In this review, we have focused on the achievements of microfluidic technology in recent decades and introduced the selection of key materials for microfluidics, as well as the characteristics and fabrication methods of microfluidic biomaterials. At the same time, we have specifically introduced the method of using 3D bioprinting to fabricate organs-on-chips. We used this example to introduce the broad applications of microfluidic biomaterials in the medical field. Finally, we have summarized the achievements in the development of microfluidic biomaterials to date and provided perspectives on their future opportunities and challenges.

## 2. Materials for Microfluidics Devices

The correct choice of different materials plays an important role in the development of microfluidic technology. The development of new materials for devices with robust and reliable performance and functionality is an essential step for the stable development of microfluidic technology. Recently, various materials have been developed for manufacturing microfluidic devices, initially glass and silicon, and later polymers and paper [56,57,58,59]. In the field of inorganic materials, in addition to silicon and glass, co-fired ceramics and in vitro ceramics are also widely used in microfluidic devices. Polymer materials can be divided into two subcategories: thermosetting materials and thermoplastic materials. Both types of materials exhibit mechanical properties ranging from rigidity to elasticity and provide a wide range of physical and chemical surface properties through adaptable formulations and abundant chemical modifications. Finally, paper microfluidics is an emerging technology based on patterned methods. The specific principle is that the device drives the liquid through capillary action through core suction in the cellulose matrix, making it one of the most promising materials today [60,61,62].

### 2.1. Inorganic Materials

**Figure 2:**
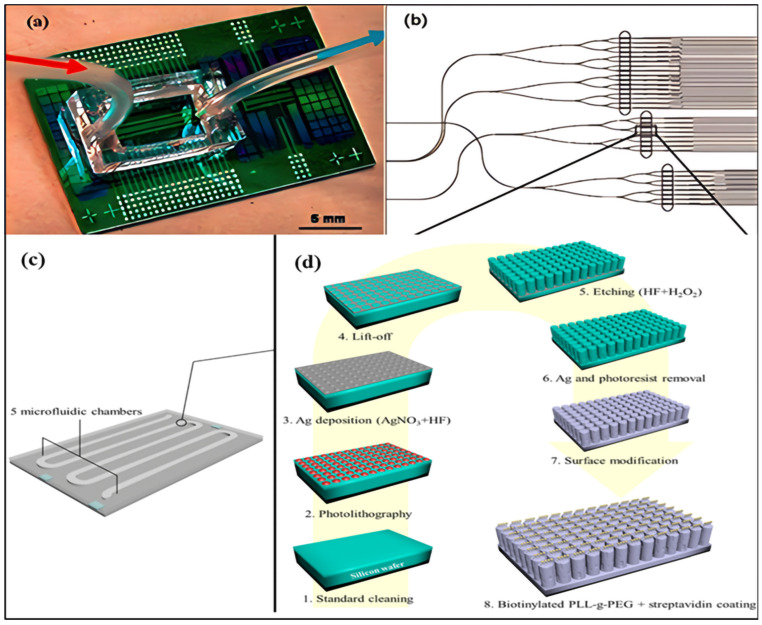
(**a**) Image of silicon nanowire device array chip. Integrated with a microfluidic technology system, the fluid is deposited into the acrylic acid well through the inlet pipe on the left (red arrow) and discharged from the outlet pipe on the right (blue arrow) to achieve fluid exchange (reprinted from Ref. [66], Copyright 2013, American Chemical Society). (**b**) A schematic diagram of waveguides and microcantilever array layout on the die (reprinted from Ref. [66], Copyright 2013, American Chemical Society). (**c**) An exemplified coral chip with 5 microfluidic chambers (reprinted from Ref. [73]). (**d**) Manufacturing process diagram and specific steps of the coral chip chamber surface (reprinted from Ref. [73]).

Inorganic materials for microfluidic devices can be divided into the following three categories: silicon, glass, and ceramics. Silicon was the first material used for microfluidics. In the mid-1980s, the integration and development of disciplines such as microelectronics and bioengineering led to the birth of microfluidic technology, which is one of the branches of fluid processing. Due to their excellent surface stability and usability, silicon and glass were used as substrate materials for microfluidic devices in the early days [63,64,65]. Although these devices, typically made of glass, silicon, or quartz, are gradually being replaced by cheaper polymer devices, they are still widely used. Currently, dry/wet etching or metal/insulation deposition methods are mainly used to manufacture silicon and glass devices [66,67,68]. Due to its high modulus of elasticity (130–180 GPa), silicon is not suitable for making active fluid components such as valves and pumps, or it may result in an overall brittleness of the part. Silane group (-Si-OH)-based silicon surface chemistry has also been well developed, and large-scale surface biochemical modifications can be accomplished through silane chemistry, such as reducing or improving cell growth through surface chemical modification [69,70,71]. Silicon is transparent to infrared light, but not within the visible light range. This characteristic may lead to serious problems and limitations, especially for optical detection methods based on biological fluorescence and direct fluid imaging. It may also face this challenge in typical fluorescence detection or fluid imaging for embedded structures. The use of transparent materials such as glass or polymer sealing in hybrid devices is the most effective solution for silicon channels and is bringing a renaissance to silicon-based detectors in microfluidic systems. In addition, to overcome the abovementioned barriers to using silicon in optical and electrical microfluidic devices, Diego Monserrat Lopez et al. first introduced silicon-on-insulator (SOI) technology for microfluidics in 2023 [72]. Applications for silicon-based detectors for microfluidic systems have ranged from nanowires for label-free cardiac biomarker detection (Figure 2a), to Si microcantilevers (Figure 2b), to silicon-based microfluidic coral [73] (Figure 2c,d).

**Figure 3:**
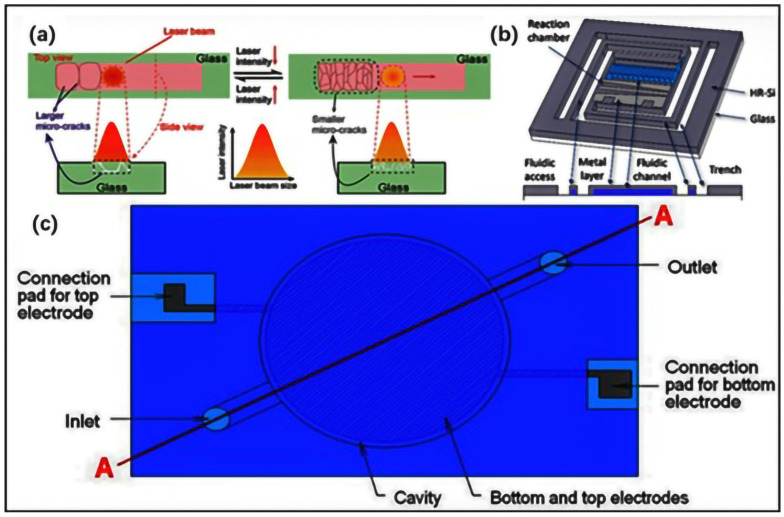
(**a**) The process of laser ablation on the surface of glass. A lower density will result in larger microcracks, which are suitable for rapid core suction and require higher energy in this case. When the energy deposition rate decreases, smaller microcracks and more of them will form, which are suitable for filtration (reprinted from Ref. [74]). (**b**) Three-dimensional and cross-sectional views of the reaction chamber on high-resistivity silicon (HR Si) chips using glass-covered microfluidic channels (reprinted from Ref. [75]). (**c**) The layout of a 3D LTCC-based ceramic structure for liquid dielectric heating (top view) (reprinted from Ref. [79]).

Based on the widespread attention given to silicon, glass has become the preferred substrate due to its excellent performance, especially in the manufacturing field of lab-on-a-chip fabrication. Due to the large elastic modulus of glass, which depends on its composition, the active components, such as valves and pumps, require mixing devices. As an applied material, glass requires mature processes such as ultraviolet lithography and chemical etching for microfabrication. Manuel Ochoa et al. presented a laser-treated glass platform for rapid wicking-driven transport and particle separation in bio-microfluidics in 2019 [74] (Figure 3a). Silicon glass technology is also applied in resonant and non-resonant single-layer microwave heaters for continuous flow microfluidics. As shown in Figure 3b, when a liquid passes through a microfluidic channel covered with glass, only the microwave heater above the reaction chamber will cause local microwave heating, but it will not heat the surrounding liquid on the fluid chip [75].

In the past few decades, ceramic microsystems, with their unique material properties and wide application potential, have become an important research direction in the field of microsystems and are especially popular in applications related to mechanical, thermal, electrical, and chemical properties [76,77,78]. A typical example is a microreactor with chemical, fluid, heating, and other functions [79,80]. Low-temperature co-fired ceramic (LTCC) was recognized as a very suitable material for their fabrication and was the most common method used for manufacturing ceramic microfluidic platforms, which is an alumina-based material in the form of a laminated board, which is patterned, assembled, and then fired at high temperatures [81,82,83]. Compared with silicon and glass platforms, ceramic microfluidic platforms are sealed monolithic microfluidic platforms with uniform surface chemical and physical properties. Based on the LTCC-based ceramic microfluidic system, Kostja Makarovǐc et al. designed a novel application of RF dielectric heating in 2021that could enable the miniaturization of microfluidic systems in many applications [79] (Figure 3c).

### 2.2. Polymers

**Figure 4:**
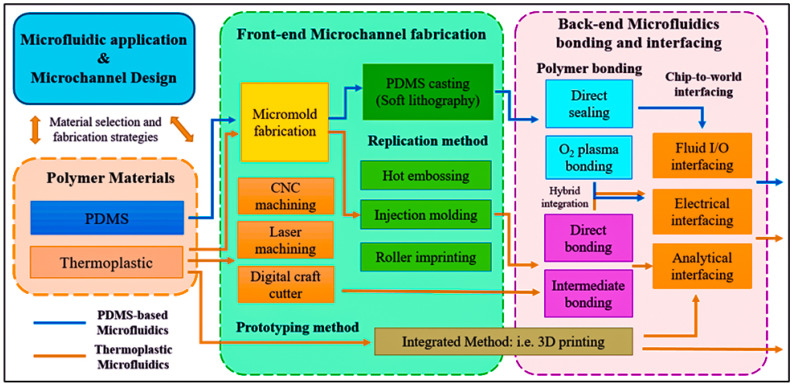
Polymer microfluidics fabrication process chart. The blue line indicates the PDMS-based microfluidics fabrication procedure, and the red line indicates the thermoplastic microfluidics fabrication procedure (reprinted from Ref. [108]).

Compared with inorganic materials such as silicon and glass, polymers are very inexpensive materials with various excellent material properties that can meet the personalized application requirements of disposable biomedical microfluidic devices and have many promising applications. Polymer materials provide cost-effectiveness and disposal advantages for manufacturing microfluidic devices. Among them, PDMS and thermoplastic are the two most commonly used main polymer materials in microfluidics [84,85,86,87,88]. Figure 4 shows the manufacturing of PDMS and thermoplastic microfluidic devices, mainly divided into two methods, as follows: front-end polymer microchannel manufacturing and back-end microfluidic bonding.

In thermoset materials, PDMS has been maturely applied in the vicinity of microfluidic chips and cell-based microfluidic devices. However, PDMS also has its drawbacks. Due to its softness, the channel deforms under applied flow pressure, resulting in variability and uncertainty in channel size. This makes the soft lithography method flawed in rheological research. Fortunately, the selective-laser-induced etching (SLE) method designed by Noa Burstein et al. in 2019 provides a potentially more comprehensive solution to the problems encountered in soft lithography.

**Figure 5:**
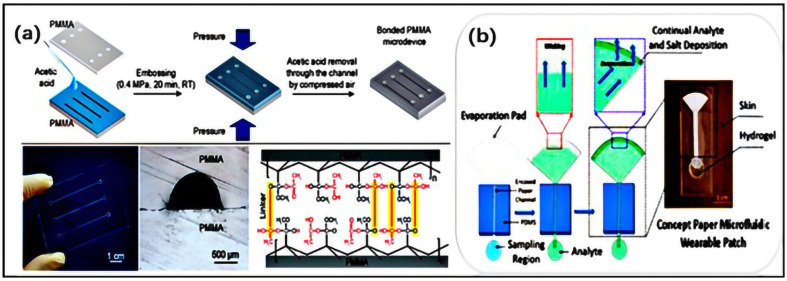
(**a**) The overall procedure of bonding two PMMA substrates with pressurized acetic acid at room temperature. After acetic acid and pressure treatment, chemical bonds will form between the two PMMA substrates in the cross-section of the bonded PMMA microdevices and microchannels, achieving the effect of overall bonding (reprinted from Ref. [122]). (**b**) The working principle of sweat collection in a paper osmotic microfluidic device (reprinted from Ref. [121]).

It is obvious that PDMS, even with outstanding performance, is not a perfect material. Therefore, the intensive use of thermoplastic materials has been increasing in recent years. These materials can be used for rapid prototyping manufacturing technology in the biotechnology industry. The typical thermoplastic materials used for microfluidics, such as PS [89,90,91], PMMA [92,93,94,95], and PET [96,97,98,99], have good transparency and solvent compatibility and low absorption of small molecules. According to the properties of thermoplastic materials, during the hot forming process, the thermoplastic sheet is heated and softened, maintaining a solid state (thermoelastic state) without losing the continuity of the material [100,101]. Thermoforming can form 3D structures, but the processing time is relatively long. Recently, thermoplastic materials have been carved through direct processing methods, including laser ablation and mechanical micro-milling, as mentioned above [102,103,104,105,106]. For example, Liu et al. proposed a plasma-assisted thermal bonding method to reduce the bonding temperature, which has been applied to PMMA microfluidic chips integrated with sealed and metal microelectrodes [107] (Figure 5a).

### 2.3. Paper

Paper is a promising microfluidic substrate. Paper-based microfluidics is an emerging microfluidic technology used in devices made of paper or other porous membranes to precisely manipulate small amounts of fluid through capillary action [109,110,111,112,113,114,115]. One of the applications of great interest is the paper-based microfluidic chip. It is an emerging microfluidic analysis technology platform with advantages such as low cost, easy processing, convenient use, and portability. Paper-based microfluidic chips use paper as the substrate, also known as microfluidic paper analysis devices. Paper-based microfluidic chips have attracted widespread interest in the application of flexible biosensors, which can be worn as wearable technology in the human body. Lightweight and flexible materials such as paper can greatly improve the complexity and high manufacturing costs of electronic device structures [116,117,118,119]. In recent years, the rapid improvement of paper-based equipment manufacturing and modification technology has led to the development of complex sample pretreatment. For example, Shay et al. developed a wearable paper-based laboratory platform with a capillary evaporation transport function for analyzing sweat samples. As shown in Figure 5b, a typical paper-based sample collection and storage device consists of three main parts, as follows: an area for storing sample fluids; closed paper channels for transporting sample fluids and paper pads with large surface areas; and an area used to continuously drive the sample fluid through the device under the action of evaporation [120,121].

## 3. Microfluidic Biomaterials

With the advancement of technology, microfluidic technology has undergone tremendous development and has been widely applied in the field of biology. Microfluidic biomaterials combine microfluidic technology and biomaterial science, with the core of designing, preparing, or regulating the physical and chemical properties of biomaterials through micro/nanoscale fluid manipulation technology to achieve precise control of their performance [16,123,124,125]. The channel width in microfluidic biomaterials requires more precise accuracy than millimeter level, therefore, microfluidic biomaterials are porous at multiple length scales, where one characterizes the channel width and the other characterizes the average pore size of the biomaterial block. The most common microfluidic biomaterials are hydrogels, such as alginate or type I collagen [126,127,128,129].

### 3.1. Features of Microfluidic Biomaterials

Compared with traditional biomaterials, functional biomaterials synthesized with microfluidics have superior performance and properties due to their controllable morphology and composition, demonstrating enormous advantages and potential in the fields of biomedical, biosensing, and tissue engineering [17,130,131]. Excellent microfluidic biomaterials must meet both the traditional constraints of biomaterials and the specific constraints of microfluidic mass transfer implementation. In other words, these materials first need to have a suitable microstructure, which can not only form a pressure-sealed fluid structure but also have high permeability for the diffusion of large and small solutes. The second constraint requires the material to have inherent low permeability to pressure-driven flow and to form a seal with itself and external pipelines. The third characteristic is crucial, as the selected material needs to allow for solute diffusion exchange between microfluidic flow and most materials.

According to functional and application requirements, microfluidic biomaterials can be divided into the following categories: as the most commonly used biomaterials, hydrogels are characterized by high water content and good biocompatibility and are commonly used in cell culture and tissue engineering; particles can be used for drug delivery, cell encapsulation, and biosensing; fiber materials are prepared using microfluidic spinning technology for tissue repair and filtration; and thin film materials can be used as coatings or separation membranes for microfluidic chips [17,130].

### 3.2. Preparation Method of Microfluidic Biomaterials

The methods for engineering microfluidic biomaterials can be grouped into categories that reflect both the patterning method itself and the types of microfluidic geometries that are achievable. Overall, the most common methods for manufacturing microfluidic biomaterials currently are divided into the following three categories: micro-molding methods, light-based methods, and 3D printing methods.

#### 3.2.1. Micro-Molding Methods

Microfluidic biomaterials can be formed by micro-molding of the material against a pre-patterned template. Microfluidic chip preparation technology utilizes microfabrication technology to construct microchannel networks on chips, achieving high-throughput preparation and screening of biomaterials. This method can precisely control the cellular microenvironment for studying cell behavior, drug screening, and tissue construction. The first, and most commonly applied, strategy is based on the micro-molding of hydrogels. The hydrogels used as microfluidic biomaterials have unique advantages. They are composed of hydrophilic polymer networks, naturally porous, and have a high moisture content, which can be used as a simulation of an extracellular matrix [132,133,134]. Therefore, the microfluidic gel obtained is very suitable for use as scaffolds for engineered living tissues. At the same time, the surface of hydrogel materials is soft, which can reduce cell damage and immune rejection. This approach is amenable to the formation of gels that contain single channels or planar networks.

**Figure 6:**
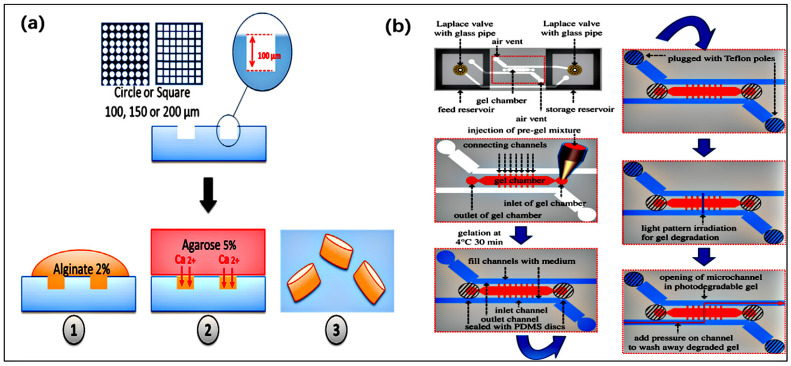
(**a**) Introduction to the morphology of microencapsulated alginate particles. The particles are produced by pouring a sterile alginate solution onto PDMS micromodels of corresponding size (diameter or side length of 100, 150, and 200 μm) and then using agarose gel loaded with 100 mM CaCl_2_ for ion crosslinking (reprinted from Ref. [137]). (**b**) The preparation of photodegradable hydrogel in the microfluidic perfusion culture device and the process of manufacturing microchannels in the photodegradable hydrogel (reprinted from Ref. [143]).

Micro-mold is also a key technology for manufacturing polymer microfluidic devices. For microfluidic devices based on thermoplastic materials, micro-mold is used to replicate microchannels through hot pressing or injection molding. For PDMS-based microfluidics, micro-mold in soft lithography is an important step in casting PDMS microchannels from molds. Currently, SU-8 micro-mold is the most commonly used method for manufacturing thermoplastic or PDMS microfluidic devices, as it is easy to operate and has lower facility costs compared to other micro-mold manufacturing methods [135]. For example, alginate micro-forming is performed using PDMS micro-molds through soft lithography. The operation steps are as follows: pour PDMS onto a silicon wafer with a positive pattern formed by SU-8 photoresist lithography and cure it at 60 °C using the RTV615B catalyst kit in a mixing ratio of 10:1 [136] (Figure 6a). To demonstrate the feasibility of using cell-friendly micro-forming technology to generate alginate-based particles, Fabien Nativel et al. employed this micro-forming method, which can produce calibrated microparticles that can be easily adjusted by changing their shape and size, thus facilitating the selection of appropriate mold sizes in their research [137]. However, there are potential issues with the adhesion between SU-8 resin and silicon substrate, and the SU-8 layer may peel off during the demolding process, which limits the lifespan of the micro-mold. An effective alternative to address this drawback is the use of polymer-based micro-forming, which has already been applied in the field of microfluidic chip manufacturing [138].

As one of the traditional techniques used for the preparation of microfluidic biomaterials, although the technology of the micro-molding method is more mature, the shortcomings exposed in terms of scalability and reproducibility still cannot be ignored. Since the micro-molding method relies heavily on pre-fabricated molds, the design flexibility is limited by the need to recreate the molds if preparing microfluidic organisms with different structures. In addition, the molds used will inevitably produce wear and tear, such as channel size deviation due to elastic deformation in PDMS molds, which largely affects the number of times the molds can be used. Effective solutions regarding these issues in micro-molding methods are still a serious challenge.

#### 3.2.2. Light-Based Methods

Light-based methods are a research approach that combines optical and microfluidic technologies for manipulating and analyzing biological samples at the microscale. The main advantage of light-based methods is high degrees of freedom, and even the ability to create complex microfluidic geometries such as branching structures or 3D networks. Traditional light-based methods can be divided into two categories: photodegradation and photopolymerization [16,139].

Photodegradation is a subtractive method used for manufacturing microfluidic biomaterials, in which light is used to remove the material to form channels. Therefore, the function of this material is similar to that of a positive photoresist. In most cases, photodegradation requires irradiation of blocky transparent hydrogels at a wavelength suitable for a specific photosensitive part of the hydrogel skeleton [140,141,142]. The main application of in vivo methods is to construct dynamic microscale concentration gradients through spatial control to provide a more realistic research environment. This method is particularly suitable for synthesizing gel, and gel made of polyethylene glycol (PEG) and its derivatives are widely used. However, because PEG itself lacks the adhesive part that allows cells to grow well on the block gel or channel surface, the use of photosensitive PEG gel is doomed to be very limited. Shinya Yamahira et al. reported a new method to construct dynamic microscale concentration gradients around cells in a step-by-step manner in micropatterned hydrogels. They innovatively used DBCO-PC-4armPEG (dibenzocyclooctyl terminated photosoluble four-arm polyethylene glycol) to prepare photodegradable hydrogel, which is a clickable cross-linking and photosoluble PEG. As shown in Figure 6b, in the microfluidic perfusion culture device, cells are encapsulated in photodegradation hydrogel, and then the perfusion microchannels are produced in the hydrogel through the photodegradation of micro patterns [143].

**Figure 7:**
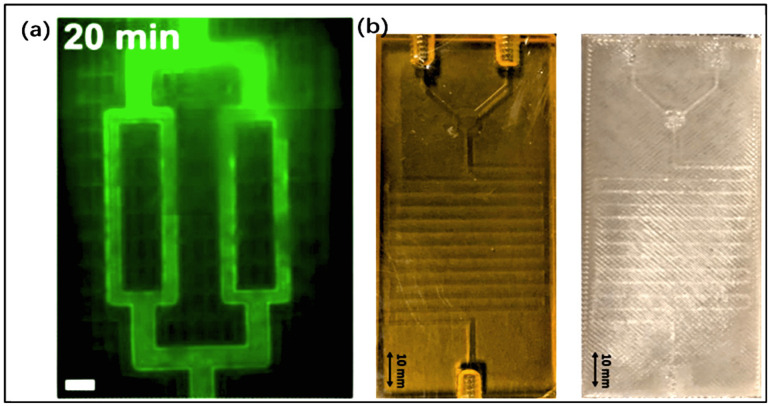
(**a**) An application example of photopolymerization is the citric-acid-based biodegradable elastomer containing a microfluidic network after perfusion with fluorescein solution in a microfluidic channel (reproduced from [16]). (**b**) Schematic diagram of 3D-printed microfluidic chips using SLA and FDM with a scale of 10 mm (reprinted from Ref. [165]).

Compared to photodegradation, photopolymerization is significantly different. Photodegradation is applied to initially crosslinked rigid gel, and the broken bond is limited to the area that has absorbed enough light. However, photopolymerization is applied to liquid precursors and then selectively crosslinked. Currently, light-based methods are often combined with 3D bioprinting. With the development of various light-based 3D bioprinting technologies and the emergence of innovative strategies, this method is widely used in vascular tissue engineering. Light-based 3D bioprinting utilizes the unique advantages of light, including high resolution, rapid solidification, multi-material adaptability, and tunable photochemistry, to provide revolutionary solutions to the challenges faced by vascular tissue engineering [144,145,146,147]. In the process of photopolymerization, biomaterials act as negative photoresists and are constructed layer by layer to form channels. In this method, when the liquid is vertically translated, a 3D structure is generated by scanning a laser on the surface of the photosensitive liquid resin. Due to the appropriate wavelength sensitivity of photosensitive groups, photopolymerization has also been used in biodegradable elastomers to form microfluidic channels (Figure 7a). The current bioprinting technology used for manufacturing live tissue models includes VAT photopolymerization [148]. Vat photopolymerization is a 3D printing technology based on photopolymerization resin. Compared to soft lithography, VAT photopolymerization has many advantages in manufacturing microfluidic devices. It not only has high precision and is suitable for manufacturing complex microfluidic channels and fine structures, but also has good compatibility and can be combined with other technologies such as soft lithography. However, material performance limitations and post-processing requirements still pose challenges for VAT photopolymerization [149].

#### 3.2.3. Three-Dimensional Printing Methods

Three-dimensional printing microfluidic technology is an advanced technology that utilizes additive manufacturing methods to manufacture microfluidic devices. It has the advantages of rapid prototyping, flexible design, and low cost, allowing for a high degree of freedom in the engineering design of biomedical devices [150,151,152,153,154]. Nowadays, 3D printing technology provides higher flexibility, faster prototype development speed, and lower cost for the design and manufacturing of microfluidics. Three-dimensional-printed microfluidics have received widespread attention due to their various advantages [155]. The 3D printing method for specific types of applications depends on the selected material type, material compatibility, material availability, size, resolution, yield, speed, and the way the final object is sliced [156,157,158].

Three-dimensional printing is also superior to traditional PDMS micro-molding in the manufacturing of microfluidic chips, especially in terms of design flexibility, manufacturing speed, and cost-effectiveness. Three-dimensional printing increases structural complexity by bypassing mold manufacturing and labor-intensive processes, greatly reducing manufacturing time and costs [159,160,161]. Moreover, with the maturity of digital light technology (DLP), water-soluble thermoplastic materials (such as ACMO) can be used as sacrificial molds that can rapidly manufacture complex structural molds and reduce post-processing steps, which not only greatly improves productivity, but also supports the repeated printing of multiple materials [162,163]. Therefore, 3D-printed microfluidic chips can be easily designed and fabricated, and a personalized custom design can produce nanomedicines at low cost. However, due to their complex geometric shape, the application of 3D-printed microfluidic chips is still immature in the manufacturing of nanomedicines [164]. To fill this gap, Aytug Kara et al. demonstrated that the encapsulation of nifedipine within polymer nanoparticles can ensure critical quality manufacturing properties using FDM and SLA 3D-printed microfluidic chips [165] (Figure 7b). They demonstrated that microfluidic chips pave the way for a simple, cost-effective, and scalable continuous process for manufacturing nanomedicine. Overall, the micro-molding method is suitable for small-scale high-precision needs in academic labs, but 3D bioprinting is more advantageous in terms of complex structural design, large-scale production, and dynamic functional integration. In the future, the preparation process of microfluidic chips can be optimized by combining the advantages of both of these through a hybrid strategy, for example, using PDMS molds to fabricate the basic channels and then adding vascularized structures through bioprinting.

As mentioned above, 3D bioprinting has been widely used to fabricate vascularized tissue constructs for disease modeling and tissue regeneration [155,166]. Specifically, light-based 3D bioprinting technology has attracted close attention and has become a breakthrough technology in this field. Due to its advantages of being digitally processable, cell-friendly, and having the ability to enhance other 3D printing technologies, various light-based 3D bioprinting methods have been developed to manufacture in vitro vascular disease models, tissue-engineered blood vessels, and vascularized tissue/organ transplants to meet the higher demands of clinical medicine [146].

#### 3.2.4. Other Methods and Future Trends

The three most common methods mentioned above often face more limitations in terms of suitable material types and/or microfluidic geometries, thus providing ease of use and the ability to quickly and conveniently generate microfluidic channels. Table 1 compares the above three methods in terms of variable restrictions, advantages, and disadvantages.

The discharge method has been used to create fractal vascular networks in degradable polymers. This method is excellent for generating large 3D branch networks in one step, although the cost is the variability of network geometry. In addition, as a better alternative method in non-photolithography, viscous fingering is the one that has seen the most application. The biggest advantage of this method is its unparalleled simplicity, but the problem it is currently facing is the lack of geometric generality.

Each of the above methods has its own unique advantages, but the range of applications is very limited. In order to compensate for, or even eliminate, the inherent shortcomings of each method, we believe that future trends in methods for preparing microfluidic biomaterials will undoubtedly require the incorporation of artificial intelligence. Currently, the combination of microfluidics in deep learning (DL) is a very innovative approach with broad prospects in the field of smart healthcare. Deep learning is particularly good at dealing with the field of big data and high-dimensional data; in addition, through its powerful pattern recognition, prediction optimization, and real-time analysis capabilities, it can significantly improve microfluidic system design, manipulation, and data processing efficiency [167,168]. For example, the combination of microfluidics with advanced technologies such as artificial intelligence has facilitated the development of wearable microfluidics, an extraordinary combination that permits high-throughput analysis of small sample sizes and advanced data processing, leading to potential breakthroughs in cell sorting, biomolecular analysis, and more [169].

## 4. Design of Organ-on-a-Chip with 3D Bioprinting Techniques

As an advanced platform that utilizes microfluidic technology and biotechnology to simulate human organ functions on chips, microfluidic-based on-chip tissues can simulate the microenvironment of human organs by constructing microchannels and cell culture chambers on the chip. The organ-on-a-chip (OoAc) platform is an innovative microfluidic device with a microscale architecture for microfluidic tissue culture. It can simulate the structure and function of human organs at the microscale, achieving the goal of repeatedly mimicking biological phenomena. These systems integrate live cells, tissues, and biomaterials into a chip-sized platform to replicate critical physiological and pathological processes, making them highly likely to become the primary preclinical testing method [37,170,171,172,173,174,175].

**Figure 8:**
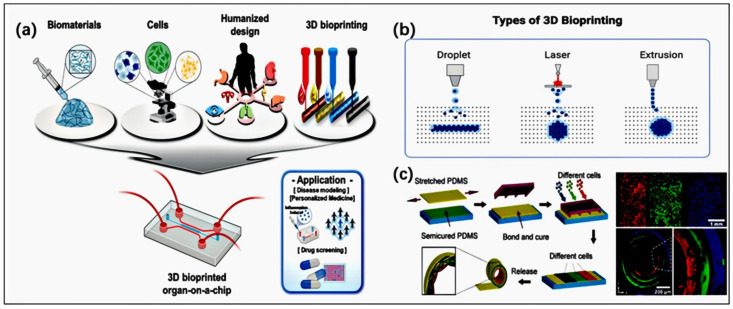
(**a**) Considerations and applications for organ-on-a-chip fabrication (reprinted from Ref. [176]). (**b**) Three-dimensional bioprinting process and types of bioprinting (reprinted from Ref. [188]). (**c**) The application of a microfluidic platform in the vascular field adopts the strategy design of a stress-induced rolling membrane to manufacture tubular structures (reprinted from Ref. [194]), Copyright 2019, Advanced Materials.

Traditional manufacturing methods, such as photopatterning, lithography, soft lithography, and self-assembly, have been used for several years to fabricate microfluidic devices and organ-on-a-chip platforms. It is worth noting that 3D bioprinting has become a promising method for manufacturing biochips. When manufacturing organ-on-a-chip devices, multiple factors such as materials, design, biocompatibility, manufacturing accuracy, and functional implementation must be comprehensively considered. These include the following: selecting appropriate biomaterials to simulate extracellular matrix (ECM) and provide support for cell growth and differentiation; and selecting the cells that make up the target organ; combining humanized design to achieve the main function and structure of the organ; and using appropriate biomanufacturing methods to construct tissue-specific environments [176] (Figure 8a).

### 4.1. Types of 3D Bioprinting

Compared with traditional manufacturing techniques, 3D bioprinting is an innovative and promising biomanufacturing strategy. Three-dimensional printing tools allow for the deposition of living cells wrapped in biological materials during the manufacturing of complex 3D structures, featuring high precision, a high degree of freedom, and high throughput. These advantages make it possible for bioprinters to establish systems that simulate the human microenvironment in a more suitable way than animal models and current 2D cell culture environments, thereby improving the accuracy of results and the clinical application of OoAC [177,178]. The identified technologies include extrusion-based techniques, such as inkjet 3D printing and photopolymerization 3D printing [179] (Figure 8b).

Extrusion-based technology is the most common 3D printing technique. Extrusion-based 3D printing technology constructs three-dimensional structures by extruding materials (such as thermoplastic, bioink, or composite materials) through nozzles and stacking them layer by layer, such as in melt deposition modeling or biological drawing [180]. This system applies specific pressure to the syringe nozzle, depositing viscous biomaterial to form a continuous liquid bead. As the extrusion head or stage moves along the *z*-axis, the next layer is sequentially stacked on top of the previously deposited layer, resulting in the creation of a 3D biological structure [181]. Control over the amount of extruded biomaterial is achieved by adjusting the pneumatic or mechanical pressure, nozzle size, or nozzle speed along the *x*- or *y*-axis. In this method, hydrogel is the main method for organ-on-a-chip applications. It is mainly manufactured through the process of nozzle extrusion and deposition on a printing bed. However, this method can be limited by the diameter of the nozzle, as well as the rheological properties of the material, such as when the viscosity of the ink leaving the nozzle has an effect on the resolution of the print; and, when the nozzle changes direction, the high viscosity of the ink will deform the print [182]. At the same time in the printing process shear stress leads to cell damage, making it difficult to print the fine structure. Therefore, the actual resolution of the extrusion method is relatively large. Although the equipment required for the extrusion method and the cost of the bioink is not high, the need for frequent nozzle changes and calibration of the pressure system increases the long-term costs. Overall, the extrusion method is suitable for bone/cartilage repair or large volume tissues.

Inkjet bioprinting is the earliest 3D bioprinting technology. Similar to traditional 2D inkjet printing, this is a non-contact bioprinting method. By using piezoelectric or thermal-driven nozzles, biological ink droplets can be divided into a series of microdroplets and accurately sprayed onto the substrate located at the top of the electronic control platform, forming a three-dimensional structure containing cells [183,184,185,186,187]. The operation of droplet-based bioprinting is similar to 2D inkjet printing, suitable for creating complex multicellular structures, but it requires the ejection of bioink with appropriate low viscosity from the nozzle [188]. However, it is the low viscosity inks that limit material choices and make it difficult to achieve precision resolution at the single-cell level. This droplet-based approach typically uses various methods, including electric heating bubbles, valve-controlled pressure pulses, and piezoelectric actuators, to deliver controlled cell or biomaterial droplets. Plaster-based inkjet printing involves printing inkjet materials composed of low-viscosity droplets in continuous layers and can print complex shapes in polymer, metal, and ceramic objects. Once the material solidifies, the printing plate will descend, and the next layer will be printed. It has the advantage of being environmentally friendly and is well suited for large-scale high-throughput screening scenarios, such as drug screening microarrays or skin tissues [189].

Laser-assisted bioprinting uses pulsed laser energy to create droplets containing cells and deliver materials. This printing method utilizes laser pulses at high speed; therefore, high-density cell droplets can be printed in a short time, and high-resolution structures can be created [190,191,192]. Regarding the light-polymerized 3D printing techniques, SLA and DLP were used. Stereoscopic lithography (SLA) is one of the oldest and most commonly used laser-assisted bioprinting methods, suitable for creating complex 3D shapes. On the contrary, digital light processing (DLP) is an emerging technology that mainly uses projectors to solidify photosensitive polymer liquids layer by layer, thereby creating 3D-printed objects. The digital micromirror device can independently rotate to modulate ultraviolet light and project optical patterns [163]. Although laser-assisted bioprinting boasts high resolution and good applicability to complex structures, the high equipment price and stringent requirements for photosensitive bio-resins predispose it to be less cost-effective [193].

### 4.2. Biomaterial Selection Criteria

The primary step in the design of OoAC devices is to correctly select the biomaterials that form the basis for chip system functionality and tissue manufacturing. These biomaterials are crucial, as they provide the necessary microenvironment for cells and contribute to structural support. Several key parameters merit consideration when choosing biomaterials for bioprinting, including biocompatibility, printability, stiffness, and crosslinking methods [195]. The selected biomaterials must have biocompatibility to create an environment where tissues and their constituent cells can grow. Biocompatible materials are non-toxic to cells and, therefore, do not cause damage or stress to the OoAC system, thereby affecting the accuracy of the results or models [196]. Biomaterials can be classified into two general categories: natural biomaterials and synthetic biomaterials.

#### 4.2.1. Natural Biomaterials

Natural biomaterials mainly come from animals or plants in nature, with unique physical and chemical properties and high biocompatibility, suitable for various biomedical applications [197]. Due to their high moisture content and viscoelasticity, these natural biomaterials are widely used in 3D bioprinting as cell encapsulated bioinks for manufacturing on-chip organic matter. Common examples include ensuring that cells are protected from external hazards such as contaminants in the printing space or mechanical stress when passing through printing nozzles.

Well suited to build structures for tissue engineering, collagen is a biomaterial often used in biomedical applications based on its biocompatible, biodegradable, and noncytotoxic properties [198,199,200]. Due to these favorable characteristics, collagen serves as a prominent biomaterial in various in vitro models, including organ-on-a-chip devices. Recently, in the field of collagen-engineered glomerulonephritis (GOC), Petrosyan et al. described a GOC model using COL-IV and laminin as matrix materials (the main components of the glomerular basement membrane). For the first time, human podocytes and human glomerular endothelial cells were seeded onto microfluidics to better simulate the process of the glomerular filtration barrier through selective permeation and response to nephrotoxic drugs [198]. In summary, collagen can open up new pathways to establish microstructures. Due to its strength, it can replicate the biological barrier of the entire organ, and its non-absorbable properties make it a powerful tool for drug analysis, disease modeling, and precision medicine applications [201,202]. However, collagen does come with limitations, such as poor physical properties, that may lead to the deformation of the fabricated structure and a reduction in printing resolution. To address these limitations, a recent development involves a hybrid collagen-derived hydrogel, in which the physical properties are enhanced by the incorporation of UV-curable materials. This innovative method has been actively applied in the manufacturing of organ-on-a-chip devices [176].

Gelatin is a natural polymer derived from the hydrolysis of collagen, which is a biocompatible, biodegradable, non-toxic, and easily processed material. The functional modification and new applications of gelatin are constantly emerging. In the field of tissue engineering, gelatin is usually used in a variety of systems, such as drug delivery systems to achieve sustained and targeted delivery, the injection of hydrogels, scaffolds, or wound dressing films for cell culture and tissue regeneration [203,204,205]. It has the advantage of strong water absorption, which can be used for tissue regeneration, such as nanocomposite hydrogel for wound treatment or injectable hydrogel for bone regeneration, but its thermal stability is not stable enough, and it can easily change from solid to gel according to the change in temperature [206,207,208]. In addition, due to its hydrophilic nature, gelatin is sensitive to moisture and becomes fluid above a certain temperature. This sensitivity can pose challenges to the structural stability of organs-on-a-chip manufactured using gelatin. To solve this problem, gelatin-methacryloyl (GelMA) is commonly used [209]. GelMA has the characteristics of both natural and synthetic biomaterials and has a three-dimensional structure suitable for cell growth and differentiation, which can replace the artificial basement membrane or other natural collagen hydrogels. The gelatin part of GelMA hydrogel provides an ideal biological adhesion environment for cells. Adding GelMA helps to crosslink and solidify gelatin through UV and visible light under the action of photoinitiators, while maintaining shape fidelity and stability at physiological temperatures, without affecting the biocompatibility and degradation performance of gelatin [210]. There is no doubt that GelMA has been widely used in tissue engineering as a hydrogel.

In the medical field, silk can be combined with hydrogels, films or coatings, nanoparticles, fibers, and other particles to obtain functional biomaterials in the form of 3D-printed structures, and mixtures of different materials are used to improve their biodegradability, biocompatibility, and mechanical properties [211,212,213,214,215]. Silk fibroin (SF) is widely used in the manufacturing of organ-on-a-chip devices using 3D bioprinting methods. It is primarily used in 3D bioprinting through physicochemical transformations of pure SF or by mixing it with a high-viscosity hydrogel or supplement to create an advanced SF-based hydrogel.

#### 4.2.2. Synthetic Biomaterials

However, due to the different types of natural sources, the quality of natural polymers varies greatly and requires careful sterilization and purification processes, which are quite cumbersome [216]. To compensate for the shortcomings of natural polymers in certain aspects, researchers have developed synthetic polymers to finely control the physical and chemical properties of materials and produce materials that can surpass the boundaries of naturally derived materials with minimal batch-to-batch variations. Synthetic biomaterials have the characteristics of customizable mechanical and biodegradable properties, excellent printing performance, and consistent quality [217,218,219]. Despite lacking bioactivity, synthetic biomaterials typically possess superior physical properties and rigidity compared to natural biomaterials. Consequently, they find application as a robust cell support framework in organs-on-a-chip. The combination of synthetic biomaterials with 3D bioprinting technology enables the realization of various organs in organs-on-a-chip [220]. The following will introduce several synthetic biomaterials widely used in organ-on-a-chip fabrication through 3D bioprinting.

Polylactic acid (PLA) is a novel biodegradable material widely used in biomedical applications, particularly in cardiac, dental, or orthopedic fixation devices, due to its biocompatibility. Its biodegradable properties can also be used for other implantable devices, such as surgical sutures that do not require removal. It is also used in regenerative medicine because it can stimulate hard tissue regeneration during bone transplantation and has extraordinary biological absorption capacity [221,222,223,224].

Polycarbonate (PCL) is also a very useful biodegradable biomaterial, with good compatibility with biological cells in vivo. Cells can grow normally on its scaffold and degrade into water and carbon dioxide. Under physiological conditions, it can slowly degrade within a few years, making it suitable for long-term implants such as treating bone defects, drug delivery systems, and delivery platforms for various extracellular matrix proteins or 3D scaffolds [225,226,227]. PCL has a lower melting point than other synthetic biomaterials and quickly solidifies after being sprayed from the nozzle [228]. This feature makes it suitable for constructing frameworks that directly interact with cells and support cellular structures. Therefore, PCL is commonly used for organ-on-a-chip tissue manufacturing through 3D bioprinting.

PDMS is an elastic and nondegradable material prepared by blending a curing agent and an elastomer base. PDMS has a wide range of applications in various fields, but it is mainly used in the production of various medical devices, such as particle blood analogs that stimulate red blood cells and microvalves or vascular analogs with excellent elasticity [229]. The PDMS-covered platform is highly suitable for cell cultures that require oxygen enrichment due to its excellent oxygen permeability. After curing, PDMS exhibits low shrinkage, high tensile modulus, and high thermal conductivity. PDMS also has optical transparency and the ability to easily track target particles, making it valuable for tissue applications on organ-on-a-chip devices that require these characteristics. The vascular model on the chip is based on the inherent advantage of precise control of fluid pathways using microfluidic technology. Through microfluidic channels and stress-induced rolling membrane technology, a series of tubular structures loaded with cells is used to simulate natural blood vessels. A unique strategy has emerged to generate simulated tubular structures with different types of cells deposited in different layers. As shown in Figure 8c, microfluidic channels are used to pattern different types of cells at fixed positions. After stretching, the PDMS film is covered with a semi-cured PDMS film to introduce mismatched tension. After releasing two layers of PDMS, a self-curling 3D tubular structure will be constructed.

### 4.3. Specific Steps of 3D Bioprinting for Organ-on-a-Chip Devices

#### 4.3.1. Bioprinting Process Stages

Three-dimensional bioprinting is generally divided into the following three stages: pre-processing, processing, and post-processing [188,230,231]. Pre-processing serves as the initial stage, where bioinks, cells, and CAD designs are prepared [230]. Different biological chains are selected according to the intended application, usually including hydrogels, extracellular matrix components, or other cell-specific materials. The cells are harvested, cultured, and mixed with bioink. The CAD schematic generated from specific imaging data or predefined structures provides a blueprint for bioprinters in specific aspects.

During the processing stage, various parameters such as nozzle diameter, extrusion speed, and layer height can be adjusted as needed through material deposition and organization for printing to optimize structural integrity and cell viability. Post-processing is the final step. Based on its expected functions (such as transplantation, drug testing, and even understanding pathophysiology), cells have the opportunity to fully integrate into their expected environment, differentiate, and interact harmoniously with natural tissues.

#### 4.3.2. Functional Design of Organ-on-a-Chip Devices

Microfluidic chip design achieves precise manipulation of fluids through microchannel structures, generating uniform droplets or fibers. Suitable materials need to be selected for functional design based on biocompatibility, controllability, responsiveness, scalability, and multifunctionality [232]. PCL is usually used as a platform material for an organ-on-a-chip device to prepare hydrogel, and the printing conditions need to be adjusted according to the properties of each hydrogel. The 3D bioprinting code is generated in the printing system, and the organ platform on the chip is printed. This is a simple method of manufacturing organ platforms on chips with complex designs through 3D bioprinting. The microfluidic channels are designed with appropriate internal dimensions according to different applications [233].

All printing structures are dried in a vacuum at room temperature, the position of the hydrogel in the printing platform is checked, and the printing microfluidic channel of the organic platform on the chip is analyzed to achieve the purpose of scanning electron microscopy. The required cells are prepared, labeled, and encapsulated in the hydrogel, and then printed on various platforms of organ-on-a-chip devices.

**Figure 9:**
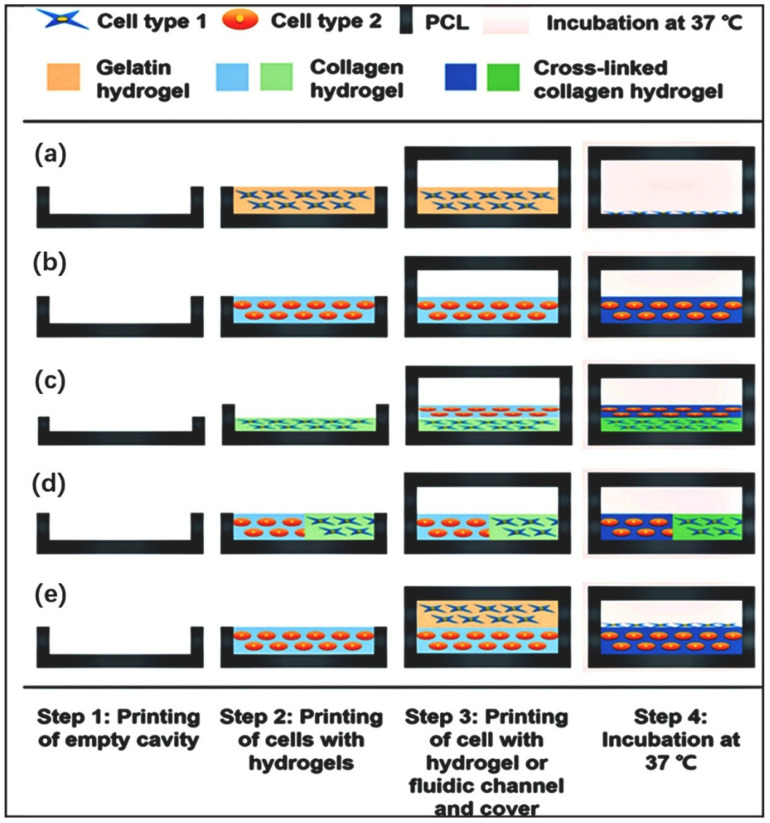
The 3D bioprinting process of organ-on-a-chip platforms for various models. In order, they are (**a**) 2D model, (**b**) 3D model, (**c**) 3D vertical model, (**d**) 3D horizontal model, and (**e**) 3D/2D model (reprinted from Ref. [233]).

Figure 9 specifically illustrates the steps of 3D bioprinting for preparing biochips. Firstly, the 3D model of the cavity is designed and prepared using CAD, and the cell types and hydrogels for the microenvironment are printed in the prepared cavity. Secondly, the sidewalls of the fluid channel are printed using a multi-layer process. By printing the shell material and crossing it above the pre-printed side walls of the fluid channel to avoid fluid leakage, the fluid channel is covered and sealed. Finally, the tube connection part for dynamic stimulation is printed. During the process of connecting the printing tube, the optimal design is chosen to ensure fluid transfer efficiency while avoiding leakage.

## 5. Medical Applications of Microfluidic Biomaterials

### 5.1. Drug Delivery Carriers

In the field of drug delivery, microfluidic technology can prepare drug carriers with uniform size and controllable drug loading [234,235,236,237,238]. By precisely controlling the morphology, size, and surface properties of the carrier, targeted delivery and controlled release of drugs can be achieved. This method has important application value in cancer treatment, gene therapy, and vaccine delivery [239,240,241]. Compared with traditional drug delivery systems, nanoparticles exhibit advantages such as increased solubility, prolonged circulation time, targeted delivery, and controlled release, making them highly stable [242]. Nanoparticles can be continuously produced in microfluidic devices, with precise control over their properties, such as size, shape, surface features, and structure, to ensure their stability. In drug delivery, microfluidic-based nanoparticles can precisely encapsulate and control the release of active agents and protect drugs from enzymatic degradation and pH changes [243]. The latest innovations and advances in microfluidics have greatly accelerated the clinical translation of nanoparticles, which are now more commonly used in the treatment of cancer and infectious diseases.

**Figure 10:**
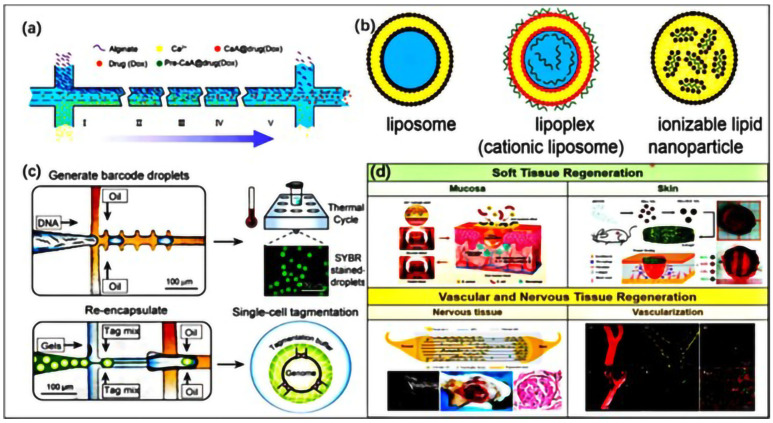
(**a**) An application example of microfluidic technology in a schematic diagram of alginate and Ca^2+^ ions diffusing into the intermediate channel and mixing to form nanoparticles (reprinted from Ref. [269]). (**b**) Lipid nanoparticles used for drug delivery. In sequence, there are single-layer liposomes with a bilayer lipid membrane surrounding the aqueous core, lipid complexes containing cationic lipids and charge-fixed nucleic acids, and lipid nanoparticles formed by encapsulating lipid/nucleic acid complexes with ionizable lipids (reprinted from Ref. [247]). (**c**) A droplet microfluidics-based system for single-cell genome sequencing (reprinted from Ref. [194]), Copyright 2019, Advanced Materials. (**d**) Typical applications of biomaterials in oral tissue engineering (reprinted from Ref. [47]).

To improve the therapeutic effect and reduce the side effects of drugs, researchers have developed different types of micro/nanocarriers using biocompatible materials, including microgel and lipid-based nanodrugs [244,245]. Micro/nanogels are three-dimensional cross-linked polymer particles with the characteristics of hydrogel and colloidal particles. Micro/nano gel has the advantages of adjustable size, large surface area, rich internal macromolecular networks, and good biocompatibility. In particular, due to their flexibility and response potential to external stimuli, they can be used as candidate materials for clinical therapeutic drug delivery. They can effectively protect encapsulated drugs from external environmental influences and transport drugs to specific tissues and cells through surface modification. Moreover, microfluidic biomaterials can simultaneously load multiple drugs, achieving combination therapy [246]. For example, alginate hydrogels have been widely used as drug carriers due to their excellent biocompatibility. Due to the remarkable pH sensitivity of sodium alginate, sodium alginate microgel can respond to the acidic microenvironment of tumors for the follow-up treatment of tumors (Figure 10a).

Lipid-based nanomedicine is a type of nanoscale drug delivery system prepared using lipid materials. It uses vesicles formed by lipid membranes to separate therapeutic agents into nanoparticles. This type of nanomedicine functionalizes lipid membranes by using ligands of various small molecules and proteins, and the particles can be easily customized for selected cells or tissues [247,248,249,250,251,252]. Although many applications of organic and inorganic nanoparticles in drug delivery have been explored, lipid-based systems remain the most researched and commercially successful nanocarriers for nanomedicine. A range of synthetic lipid constructs have been employed for drug delivery (Figure 10b). For example, nanoscale liposomes composed of lipid bilayer vesicles surrounding a water-based core, with a structure similar to that of a cell membrane, can encapsulate the water-based core containing hydrophilic and hydrophobic compounds, making them highly suitable for delivering various therapeutic agents [253].

### 5.2. Cell Analysis

Microfluidic technology has gained great attention in cell analysis due to its advantages of high throughput, high precision, and real-time monitoring. Standard procedures such as single-cell analysis, cell culture, sorting, lysis, and content separation all rely on microfluidic technology. The emerging single-cell technology is a technique that enables research and analysis at the single-cell level and has made significant progress in revealing single-cell heterogeneity [254,255,256,257,258,259]. The ability of fluid control to miniaturize size has rapidly improved with technological advancements, making microfluidic technology a leading technology for single-cell capture, preservation, and analysis. Nowadays, droplet-based microfluidic technology is widely used for single-cell high-throughput analysis. For example, Figure 10c shows a droplet microfluidic platform for high-throughput single-cell genome sequencing. Single cells are encapsulated in hydrogel microspheres, which can capture genomic DNA stereoscopically and penetrate small molecules such as enzymes and detergents [194,260]. In the fields of single-cell proteomics and genomic analysis, the use of droplet microfluidics can confine single cells to small volumes, allowing secreted metabolites to rapidly accumulate to detectable levels. This not only improves efficiency, but may also allow them to become an integrated platform for high-throughput screening [261,262]. At present, the key step in biological research is the microfluidic system used for intracellular delivery. Compared with traditional intracellular delivery methods, microfluidic systems are becoming a new possibility for revitalizing intracellular delivery due to their ability to precisely manipulate cells.

### 5.3. Tissue Engineering

Tissue engineering is an interdisciplinary field aimed at utilizing principles from engineering, materials science, and life sciences to construct, repair, or replace damaged tissues and organs [263]. Tissue engineering utilizes microfluidic chips to simulate the microenvironment in vivo, study cell behavior and tissue formation, and create 3D tissues that accurately simulate tissue function for regenerative medicine and disease modeling [264,265]. Currently, developments in organ-on-a-chip systems have increased tissue-specific in vitro models for a range of tissues/organs mimicking their primary functions [26].

As mentioned earlier, bioprinting of vascular tissue has enormous potential in tissue engineering. Microfluidic technology provides an ideal platform for reconstructing vascular chip models. The vascular network can simulate the shear stress and cyclic stretching of arterial endothelial cells in microfluidics and support metabolic activity. Compared with existing artificial tubes, microfluidic vascular chips provide enormous potential for simulating the physiological microenvironment for drug discovery and evaluation. Microfluidic vascular chips can accurately simulate the geometric structure and branching network of blood vessels and simulate the physiological functions of blood vessels by regulating fluid dynamics parameters, which reflects the reliability of microfluidic models for practical applications [194].

Oral tissue engineering utilizes cells, scaffold materials, and bioactive molecules, combined with 3D bioprinting, microfluidic technology, and bioreactors, to repair or regenerate oral tissues such as teeth, periodontal tissue, jawbone, and oral mucosa, and can also restore oral function. However, complex tissue types and precise anatomical structures pose challenges for oral and maxillofacial restoration, but biomaterials also play a crucial role in oral tissue engineering [47,266,267,268]. For the methods of repair, biomaterials are required to repair the destroyed bone tissue and guarantee a secure bond between the materials and the host bone. Also, biomaterials are used for soft tissue, vascular, and nervous tissue regeneration (Figure 10d). The multifunctionality and superior tissue regeneration ability of oral tissue engineering in morphology may lead to clinical and experimental development. In the future, with the development of personalized medicine, intelligent materials, interdisciplinary integration, and clinical translation, the application prospects of oral tissue engineering will be even broader.

## 6. Conclusions and Prospects

In this review, we present the current status of microfluidic technology. In recent years, significant progress has been made in microfluidics-based biomaterials and biodevices. We have summarized the major advances in functional design and biological applications of microfluidic biomaterials. For material selection, we went from glass and silicon at the beginning to polymeric materials, which have undergone significant development. For design methods, although traditional methods such as micro-molding and light-based methods have matured after decades of development, new methods of 3D bioprinting have recently shown more promise, especially in the fabrication of organs-on-chips. For applications, researchers can design biomaterials with precise structures and functions using microfluidic technology, which has shown broad application prospects in fields such as drug delivery, tissue engineering, and cell culture.

However, there are still many challenges that need to be addressed in terms of material properties, preparation processes, and application scenarios to promote further development in this field. Currently, there is no “perfect material”, as each material has its inherent advantages and disadvantages. Future research needs to further optimize the design and fabrication processes of microfluidic systems, improve the biocompatibility and functionality of materials, and promote their clinical applications. Meanwhile, interdisciplinary collaboration will be the key to advancing this field, and the deep integration of materials science, microfabrication technology, biology, and medicine will bring more innovation and breakthroughs to microfluidic biomaterials.

Microfluidic biomaterials are at a critical stage of transition from the laboratory to the clinic. In biomedical research, microfluidic systems have shown great potential in cell biology and organs-on-chips. Researchers are constantly using microfluidics to study biology, accelerating our understanding of our bodies and discovering the laws of nature, until organs-on-chips can replace animal experiments and even clinical trials. Specifically, the future of microfluidics in biomedicine undoubtedly belongs to the era of 3D bioprinting, which is capable of fabricating vascularized organ models with hierarchical structures, which cannot be achieved using traditional methods (e.g., soft lithography). To address scalability issues, we suggest exploring modular designs and automated production pipelines. For clinical translation, standardized biocompatibility protocols and long-term stability testing under physiological conditions are critical. At the same time, we cannot ignore the fact that the application of deep learning in microfluidic systems has shown a strong development trend, and artificial intelligence technology will gradually cover the microfluidic system in data processing, state evaluation, intelligent decision making, and automatic optimization. It can be said that the improvement of the intelligence level of microfluidic systems is unstoppable. Perhaps soon, microfluidics will be integrated into various aspects of our bodies. From drugs to tissues, from organs to our smart wearable products, microfluidics will play an increasingly important role.

## Figures and Tables

**Table 1:** Comparison between different methods used to prepare microfluidic biomaterials.

Methods	Variable Limitations	Advantages	Disadvantages	Reference
Micro-molding	Mold precision limitations, material needs thermoplastic	High yield, low cost	Prefabricated molds required, low design flexibility	[133]
Light-based	Lithography resolution, photosensitive material limitations	High precision, high resolution	High equipment costs and cumbersome processes	[139]
3D Printing	Print resolution, limited material options	Rapid prototyping, complex 3D structures can be prepared, no molds required	Resolution and speed need to be improved	[162]

## Data Availability

No new data were created or analyzed in this study. Data sharing is not applicable to this article.
